# Expression of LINE-1 elements is required for preimplantation development and totipotency

**DOI:** 10.1016/j.gendis.2025.101555

**Published:** 2025-02-07

**Authors:** Ru Ma, Nan Xiao, Na Liu

**Affiliations:** a School of Medicine, Nankai University, Tianjin 300071, China; b Tianjin Key Laboratory of Human Development and Reproductive Regulation, Nankai University Affiliated Maternity Hospital, Tianjin 300100, China

**Keywords:** Embryonic stem cells, Epigenetic, LINE-1, Preimplantation development, Totipotent, Transposable elements

## Abstract

Transposable elements, long considered genomic intruders, have been found to play significant and intriguing roles during early embryonic development based on the paradigm shift that has undergone in recent years. Long interspersed element-1 (LINE-1) is the predominant class of retrotransposons with autonomous retrotransposition capabilities in mammals and has emerged as a crucial element of preimplantation development. In this review, we elucidate the expression dynamics of key transposable elements throughout preimplantation development and their contribution to the regulation of developmental progression and totipotency. We also explore the critical function of LINE-1 activation and its rich functional reservoir, which is exploited by the host to provide cis-regulatory elements and functional proteins. Particular highlights of the widespread activities in preimplantation development of LINE-1 during multiple epigenetic modifications such as DNA methylation, histone methylation, ubiquitination, and RNA methylation. The silencing complex and RNA exosome also coordinate with LINE-1 across distinct developmental stages. Accordingly, the up-regulated expression of LINE-1 retrotransposons and their protein products plays a key role in various processes, including the opening of chromatin architecture, zygotic genome activation, aging, and age-related disorders. It may reflect an effect on totipotency and pluripotency of mammalian development. Underscoring its pivotal significance, the nuanced regulation of LINE-1 illuminates its indispensable role in orchestrating the precise coordination essential for the regulation of cellular pluripotency and the intricate mechanisms of zygotic genome activation.

## Introduction

Transposable elements (TEs), also known as “jumping genes”, are mobile DNA sequences initially identified in maize in the 1950s.^1^ TEs are found in almost all organisms, making up almost half of the mammalian genome.^2^^,^^3^ TEs can transpose within the host genome, but transposon insertions in exons are generally deleterious to the host, resulting in their elimination by natural selection. Remarkably, TEs are capable of generating significant heterogeneity and variation in gene regulation, shaping the landscape of genetic information through their ability to transpose within the host genome over evolutionary history.^4^

Retrotransposons, a subset of TEs, are notable for their dramatic contributions to shaping genomic architecture and genetic information. In humans, retrotransposons such as long interspersed element-1 (LINE-1) play a crucial role in generating genomic variety and architectural variation thereby facilitating generic innovation throughout human evolution. As early as the association between retrotransposons and pre-implantation development had not been discovered, Joanna et al found that LINE-1 elements were transcriptionally active early in pre-implantation development and that they were most highly transcribed at the 2-cell stage.^5^ Similarly, they revealed the highest degree of hypomethylation in mouse induced pluripotent stem cells in almost all TEs and the whole genome.^6^ Early embryonic development undergoes widespread epigenetic remodeling and chromatin reorganization, and consequently, retrotransposons become a support factor based on their elegant and active activities during this period. Recent research has shed light on the intricate role of retrotransposons in genome evolution and functional diversity, underscoring their importance in understanding the complexities of genetic processes. In this review, we discuss the origin, classification, and evolutionary significance of retrotransposon elements. It also discusses the regulatory roles and mechanisms of LINE-1 and endogenous retroviruses (ERVs) in orchestrating pre-implantation embryonic development, with a focus on their potential contributions to cellular totipotency.

### TEs

**Figure 1:**
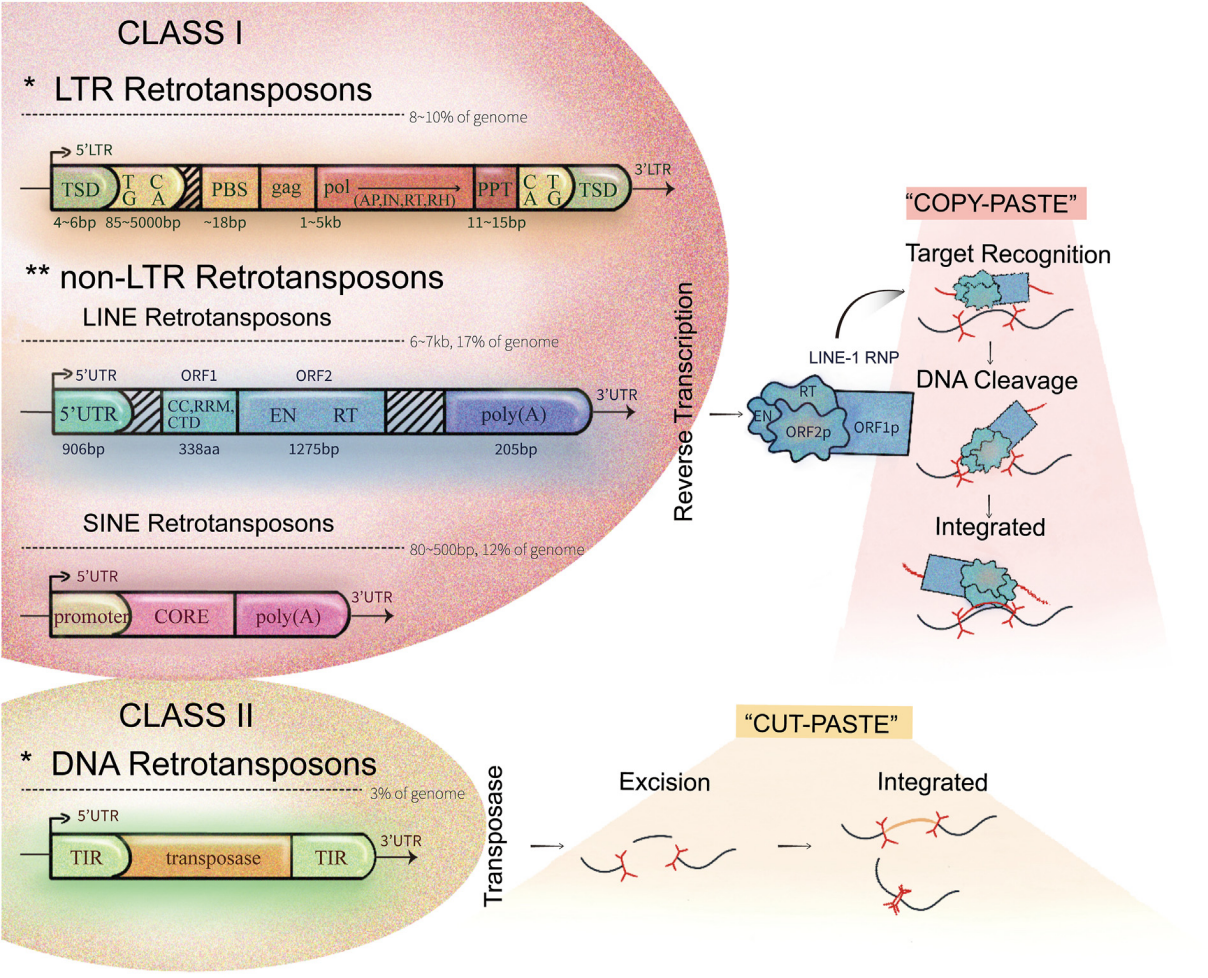
Structures and transposition mechanisms of TEs. In the nucleus, LINE-1 ribonucleoproteins, facilitated by ORF2p, initiate transposition by cleaving one DNA strand, creating 3′OH ends, followed by cleavage of both strands to generate complementary sticky ends. Concurrently, ORF2p′s transposase activity creates a gap in the target DNA, allowing excised transposons to integrate into new DNA through a “COPY-PASTE” mechanism, forming stable bonds. Conversely, DNA transposons utilize a “CUT-PASTE” mechanism mediated by transposase enzymes: transposase recognizes transposon ends, creates staggered breaks in the target DNA, transfers the transposon, and repairs the gaps, relocating the transposon. The TSD signals transposon insertion. LTRs consist of terminal repeats at both ends of the transposon, including the TG.CA box. The PBS binds complementarily to the 3′ end of certain tRNAs and serves as the initiation site for reverse transcription. Protein domains include GAG and POL, the latter containing several enzymes essential for LTR autotransposition. The PPT is a short purine-rich sequence located at the start of the 3′ LTR. UTRs contain both sense and antisense internal promoters. ORF1 of LINE-1 encodes a CC domain, an RRM, and a CTD, while ORF2 includes EN and RT domains. TEs, transposable elements; LINE-1, long interspersed element-1; TSD, target site duplication; LTR, long terminal repeats; PBS, primer binding site; POL, polymerase; GAG, group-specific antigen; UTRs, untranslated regions; CC, coiled-coil; RRM, RNA recognition motif; CTD, carboxyl-terminal domain; EN, endonuclease; RT, reverse transcriptase.

TEs are classified into two categories: retrotransposons (subclass I elements) and DNA transposons (subclass II elements), based on their mechanism of transposition.^7^ Retrotransposons are characterized by cleaving only a single strand,^8^ replicating through a “copy-paste” mechanism that does not fully extract DNA strands from the host genome.^9^^,^^10^ In contrast, DNA transposon elements require cleavage of the double strands for transposition and employ a “cut-and-paste” mechanism without the intermediacy of RNA.^8^^,^^11^ Retrotransposons are notable for encoding multiple proteins and carrying out a diverse range of biochemical activities.^8^ They are transcribed into RNA, reverse-transcribed, and reintegrated into the genome, effectively transposing the element in a copy-and-paste manner. Retrotransposons are further categorized into long terminal repeat (LTR) and non-long terminal repeat (non-LTR) retrotransposons (Fig. 1).

#### LTR retrotransposons

ERVs are the most representative of LTR retrotransposons, accounting for 10 % and 8 % of the mouse and human genomes, respectively.^12^ ERVs can be classified as class I, II, and III elements based on the sequence of their reverse transcriptase genes. Human ERVs (HERVs) are structurally similar to simple retroviruses and have two LTRs with signals to regulate stage-specific transcription. Typical ERVs contain polymerase (pol), group-specific antigen (gag), and protease (pro) genes. A small subset of retrotransposons also encode env (envelope protein), which facilitates the extracellular infectious phase, and most ERVs (*e.g.*, murine endogenous retrovirus type L/MERV-L) do not have an env gene and are incapable of horizontal transfer.^13^ Transcription of ERVs is initiated at the 5′ LTR promoter, producing mRNA that is translated into gag and gag-pro-pol fusion proteins, which are then reverse-transcribed into LTR-containing double-stranded cDNAs. ERVs eventually integrate into the host genome by ERV integrase and increase the size of the host genome.^14^ However, almost all HERVs have lost the ability to transpose in the human genome due to the loss of regulatory elements or protein-coding sequences.^14^ Nevertheless, these elements still play an important role in zygotic genomic activation during preimplantation development.

#### Non-LTR retrotransposons

Non-LTR retrotransposons are further subdivided into long interspersed elements (LINEs) and short interspersed elements (SINEs), which are repetitive short (80–500 bp) sequences that make up about 12% of the human genome and do not encode proteins.^15^ LINE-1 elements comprise approximately 17% of the human genome and are the most common autonomous retrotransposons identified in the human and mouse genomes. Due to the varying number of mobile TEs in each species, it is estimated that the mobilization rate of LINE-1 is at least an order of magnitude higher in the mouse germline than in humans.^16^^,^^17^ The human LINE-1 consists of a 5′-untranslated region (5′ UTR) containing their promoters, two open reading frame proteins (ORF1 and ORF2), a 3′ UTR, and a poly(A) tail. Specifically, ORF1 encodes a trimeric protein (ORF1p) with RNA-binding protein and nucleic acid chaperone activity, while ORF2 encodes a protein (ORF2p) with endonuclease and reverse transcriptase activities, as well as a Cys-rich domain. All three domains are essential for retrotransposition.^18^ Upon insertion, LINE-1 typically generates direct repeats of 7–20 bp flanking the sequence, which are called target site duplications.^19^ Subsequently, LINE-1 transcribes approximately 6 kb RNAs by autonomous promoters and then translates into ORF1p and ORF2p, traffics back to the nucleus, and finally forms the new genomic insertion.^20^ LINE-1 elements in mice exhibit structural similarities to those in humans; however, they are distinguished by a variant RNA polymerase II promoter structure at the 5′ terminus, and they occupy non-syntenic loci in the chromosomes.^21^^,^^22^ Mouse LINE-1 sequences can be classified into at least five structurally distinct subfamilies (V-, F-, A-, Tf-, and Gf-type). The A-, Tf-, and Gf-type maintain retrotransposition capacity,^23^ whereas the V- and F-type are inactive.^24^ Based on these marvelous features, we can learn that the retrotransposons are critical for the progression of mammalian preimplantation development, a fulcrum for reversing the reputation of LINE-1 and further elaborating its reactivation in early embryonic development.

Mice and humans share 99% of their protein-coding genes, but their regulation varies, resulting in differences between them. Transposon insertion events are generally harmful to the host and natural selection removes them from the population. Hosts have evolved stringent control programs to maintain genome stability as such TE mobilization leads to spontaneous mutations. Part of these regulatory programs are epigenetically determined, with DNA and histone modifications^25^, ^26^, ^27^, ^28^ leading to transcriptional silencing, besides other transcriptional and post-transcriptional^29^^,^^30^ defense mechanisms that have also been identified.

Some transposons have little effect on host fitness and are fixed in the population by genetic drift. In mammals, genomes have accumulated millions of retrotransposable sequences throughout evolution. Recently, scientists have begun to focus on this portion of TEs and have demonstrated that retrotransposons are transcribed more in early embryos and cells (pluripotent stem cells) than in somatic cells. Some factors regulating the elements have been identified. LINE-1 retrotransposition or activation is observed in mammalian early embryo development and pluripotent stem cells, such as embryonic stem cells (ESCs) and induced pluripotent stem cells.^5^^,^^31^

### TEs in preimplantation development

**Figure 2:**
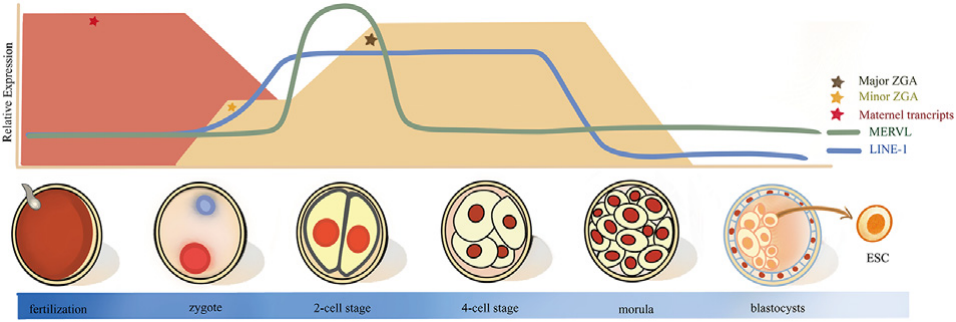
Schematic illustration of the dynamic transcription of TEs during murine pre-implantation development. Minor ZGA occurs at the one-cell stage, while major ZGA occurs at the 2-cell stage. MERV-L expression peaks at the 2-cell stage, declining until the blastocyst stage, while LINE-1 element transcription begins post-fertilization, peaks at the 2-cell stage, and persists through initial cell divisions. ZGA, zygotic genome activation; MERV-L, murine endogenous retrovirus type L.

Mammalian fertilization begins with the fusion of a sperm and an oocyte, followed by epigenetic remodeling, resulting in the formation of the totipotent zygote. The totipotent embryos then undergo cleavage divisions, reaching the 2-cell, 4-cell, and 8-cell stages before starting morphogenesis with the formation of the compacted morula and then the expanded blastocyst after implantation.^32^ The period from fertilization to blastocyst formation is preimplantation development, involving a series of pivotal events such as zygotic genome activation (ZGA), which are crucial for embryogenesis.^33^, ^34^, ^35^ The zygotic genome is transcriptionally activated through the maternal-to-zygotic transition, enabling the zygote to be reprogrammed to a totipotent state.^36^ ZGA refers to the initiation of gene expression after fertilization and the subsequent transition from maternal to zygotic control.^37^ In mice, ZGA is categorized as minor ZGA, occurring during the middle of the one-cell stage and early 2-cell stage, and major ZGA, occurring during the mid-to-late 2-cell stage.^38^ In humans, ZGA commences at the 4-cell and 8-cell stages.^39^ It is characterized by the loss of heterochromatin, DNA demethylation, and increased histone mobility.^40^^,^^41^ Notably, the activity of TEs is closely associated with preimplantation development. During ZGA, a substantial number of retrotransposons, including ERVs, LINE-1 elements, and the non-autonomous SINEs, are activated. The abundance and activity of various types of TEs during mammalian preimplantation development vary significantly, contributing to the establishment of totipotency, pluripotency, cellular plasticity, and the activation of the embryonic genome.^5^^,^^31^^,^^42^, ^43^, ^44^, ^45^ Specifically, mouse zygote and 2-cell stage embryos actively transcribe numerous retrotransposons that persist into the blastocyst stage^46^ (Fig. 2).

#### Dynamic expression of LINE-1 during preimplantation development

LINE-1 elements are transcribed soon after fertilization, with the highest transcriptional activity observed at the 2-cell stage,^5^ which persists through the first two rounds of cell division.^5^^,^^47^^,^^48^ LINE-1 activation and expression represent some of the earliest transcriptional events during ZGA at the 2-cell stage.^5^ The up-regulation of specific genes, known as “2C genes” or the “2C program” occurs during the 2-cell stage and marks the transition from maternal control to ZGA. Specifically, robust expression of 2C-specific genes such as *Dux*^43^ and *ZSCAN4*,^49^, ^50^, ^51^ high histone mobility,^40^^,^^52^ and relaxed chromatin structure^43^^,^^53^ are observed in both 2-cell embryos and mouse embryonic stem cells (mESCs). However, there are also subtle differences in the epigenetic and transcriptomic profiles between the *de novo* and *in vitro* experimental models.^54^ In ESCs, approximately 19% of pluripotent transcription factor-binding sites are located in TEs in both human and mESCs. Knockdown of LINE-1 leads to totipotency in ESCs, and 2-cell genes including well-known markers, *Zscan4*, *Dub1*, *Gm4340*, *Tcstv1/3*, and *Zfp352* are up-regulated.^21^ In human embryo development, decreased LINE-1 methylation levels have been found at the 4-cell stage, with increased expression of LINE elements at the 8-cell stage embryos (human ZGA stage).^55^ The expression of LINE-1 family elements varies at different preimplantation stages, indicating that LINE-1 elements are necessary for early developmental progress.

#### ERV retrotransposons in early embryonic development

During early mammalian embryonic development, the transcriptional activation of ERVs is a species-specific and conserved biological process.^56^^,^^57^ The earliest characterized ERVs include class II intracisternal A particle (IAP), MERV-L, and mammalian apparent LTR retrotransposon (MaLR).^12^ In the host-virus interplay, ERV activation is regulated by a multilayered network that balances ERV activation and repression, resulting in stage-specific expression of ERV during pre-implantation embryo development.^57^ It is worth noting that 2-cell-like (2C-like) mESCs also exhibit high transcriptional output on LTR elements, particularly MERV-L elements.^58^ MaLR expression is restricted to the zygote and 2-cell stage embryos.^59^ Transcripts of IAPs are derived from the parent material that degraded rapidly after fertilization. They are suppressed during ZGA and subsequently re-expressed, peaking at the blastocyst stage.^60^^,^^61^ MERV-L is expressed in cleavage-stage embryos, where it drives the expression of an enormous number of transcripts specific to ZGA and totipotency.^59^ Specifically, it is rapidly transcribed at the beginning of the S phase of the first cell cycle (8–10 h after fertilization).^62^ Down-regulation of MERV-L can result in developmental arrest at the 2-cell stage.^63^ Therefore, MERV-L is considered crucial for the embryogenesis.^58^ Strongly related *Dux* is necessary for preimplantation development^44^ which regulates MERV-L through direct binding of the motif within the LTR, affecting 25 % of minor ZGA genes in embryos and inducing 2-cell-like ESCs in mESCs.^64^ Likewise, *Dppa2* and *Dppa4* have been shown to bind to the *Dux* regulatory region to facilitate ZGA, and *GATA2* also regulated MERV-L expression in mouse induced pluripotent stem cells. Additionally, to *Dux*, *ZSCAN4* can also initiate MERV-L^64^. It is worth noting that the transcription factor binding sites located within the LTR component are species-specific, so overexpression of the human homolog *DUX4* does not activate the mouse ERVs.

HERV elements regulate and generate a variety of stage-specific RNAs in early human embryos, which indicates that expression profiles of HERV families also correspond to cell identity. HERV families, such as HERV-H, HERV-L, and HERV-K, which are associated with early embryonic development, signify an undifferentiated state. Furthermore, LTR3B and LTR14B (part of HERV-K) are enriched in the oocyte to 4-cell stage, MLT2A1 (HERV-L) is enriched in the 8-cell stage, LTR5_Hs (HERV-K) is enriched in morula, and LTR7B and LTR7Y (HERV-H) are enriched in the 8-cell and morula, respectively.^65^ Many critical ZGA-specific genes are regulated by LTR elements of ERVs.^59^ ERV expression correlates extremely well with the expression of stage-specific protein-coding genes during early development, indicating that expression profiles of HERV families may herald cell identity.^65^ Collectively, ERV expression is a common phenomenon in pre-implantation embryos that is systematically controlled and generates large numbers of co-expressed, stage-specific transcripts with potential biodiversity.

### Factors regulating the activity of retrotransposons

**Figure 3:**
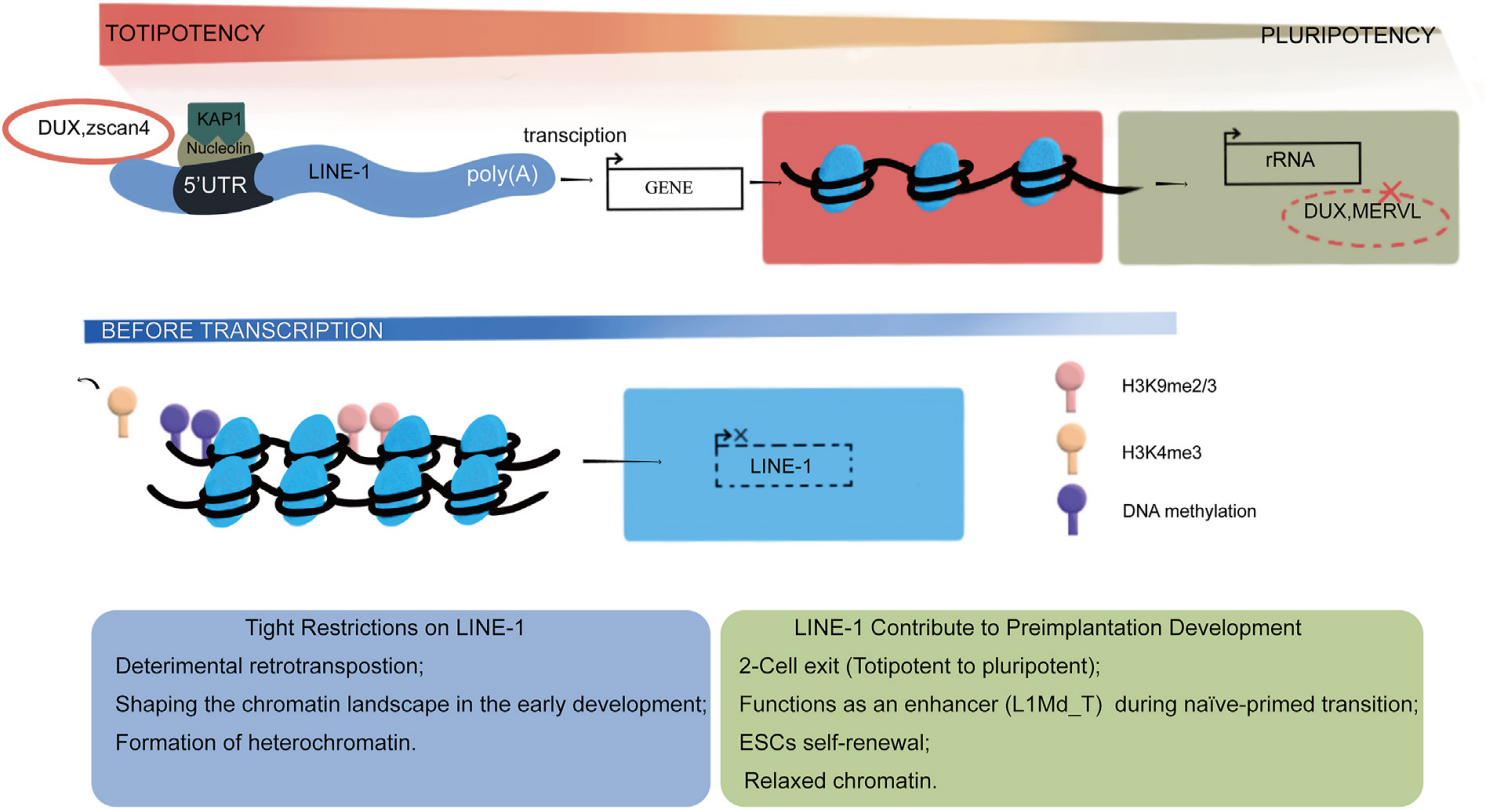
The factors regulating the activity of retrotransposons in early embryo development. Highly coordinated molecular mechanisms, including cis- and trans-acting, RNA decay, and epigenetic modifications such as histone modifications, DNA methylation, RNA methylation, *etc*., keep LINE-1 under tight control. At the same time, LINE-1 also plays a role in early embryonic development and exit from totipotency in various ways.

The stage-specific activation of retrotransposons is strictly controlled by precise epigenetic regulation in various ways, including DNA methylation, histone modification, RNA modification, exosome complexes, and post-transcriptional degradation (Fig. 3).

#### DNA methylation inhibits the activation of retrotransposon locus

DNA methylation, a critical epigenetic regulation, modulates gene transcription by adding a methyl (CH3) group to cytosines through the action of DNA methyltransferases (DNMTs).^66^ During preimplantation development, the zygote undergoes two waves of DNA demethylation. Both maternal and paternal genomes are globally demethylated after fertilization.^67^ Subsequently, 5-methylcytosine levels in the preimplantation embryo reach a relatively low level, which is increased after implantation.^34^ DNA methylation is essential for maintaining genome stability by silencing the expression of TEs.^60^ Early embryonic control of LINE-1 is an evolutionarily dynamic process, a model showed that newly emerged human-specific counterpart lineages (LINE-1Hs) are initially suppressed by DNA methylation-inducing small RNA-based mechanisms.^68^ In contrast, L1 is bound by KRAB-associated protein 1 (KAP1) and represses the heterologous promoter in human embryonic stem cells (hESCs), having entered the ancestral genome earlier in evolution.^68^ Additionally, TET1 knockdown has been shown to up-regulate L1Md_Ts in mESCs grown in 2i/LIF conditions, which depends on DNA demethylation.^69^

Taken together, DNA methylation seems to play an important but not exclusive role in the suppression of retrotransposon activation during preimplantation development.

#### Post-transcriptional degradation of LINE-1

Both the KRAB/KAP1 pathway and DNA methylation exported by small RNA-based mechanisms are involved in limiting LINE-1 in ESCs, yet function on an evolutionarily distinct set of elements.^68^^,^^70^ The typical degradation procedures for LINE-1 also include the nuclear exosome targeting-mediated nuclear degradation and “human silencing hub (HUSH)”. In brief, transcriptional and post-transcriptional mechanisms synergistically suppress the genotoxic potential of TE RNAs and flexibly regulate their activity at particular scopes and spatial scales on the genome.

RNA modification has gained recognition for its widespread and significant function. The most predominant class of RNA methylation modifications is N6-methyladenosine (m^6^A), which primarily occurs on conserved RRACH sequence motifs (in which R denotes A or G, and H denotes A, C or U) and is regulated by writer, eraser, and reader.^71^ TEs have also substantially shaped human evolution at the RNA level by regulating the expression of nearby genes.^72^

Independent reports have confirmed that the deletion of m^6^A leads to transcriptional activation, associated with increased chromatin accessibility, enrichment of specific transcription factors, and elevated histone markers. The m^6^A modification of mRNAs is catalytically generated by the m^6^A methylation transferase complex (MTC), which contains the core catalytic proteins methyltransferase like 3 (METTL3) and methyltransferase like 4 (METTL4), as well as multiple regulatory proteins, including WT1 associated protein (WTAP), vir-like m^6^A methyltransferase associated (VIRMA), HAKAI (also known as Casitas B-lineage lymphoma-transforming sequence-like protein 1, CBLL1), zinc finger CCCH-type containing 13 (ZC3H13), and RNA binding motif protein 15 (RBM15).^73^ Novelly, m^6^A is highly abundant in LINE-1 and can act as a chromatin-associated regulatory RNA (carRNA) to regulate transcription by affecting the chromatin structure at the corresponding genomic locus.^74^ Especially younger LINE-1, L1Md_F, which is utilized by either the m^6^A writer METTL3 or the nuclear reader YT521-B homology-domain-containing protein 1 (YTHDC1) to affect chromatin state and transcription in an m^6^A-dependent manner.^75^ In mESCs, knockout of certain subunits of the m^6^A writer complex (METTL3, METTL4, WTAP, ZC3H13) elevated LINE-1 abundance.^76^ This equally results in fat mass and obesity-associated protein (FTO), the first erasing protein to mediate m^6^A demethylation,^73^^,^^77^ maintains LINE-1 RNA abundance in mESCs and promotes chromatin accessibility and LINE1 gene activity.^78^ This reveals a direct correlation between carRNAs, m^6^A methylation, and chromatin status. Intriguingly, m^6^A functions in an extraordinary way using the LINE-1 5′ UTR as a hub regulating its retrotransposition.^79^ Another eraser, ALKB homolog 5 (ALKBH5), mediates m^6^A demethylation of LINE-1 in hESCs, overexpression of which inhibits the jumping function of LINE-1. Remarkably, accurately formed m^6^A clusters in the 5′ UTR of LINE-1 RNA are essential for LINE-1 retrotransposition and promote the formation of LINE-1 ribonucleoprotein, whereas the absence of m^6^A modification inhibits LINE-1 cDNA expression and the translation efficiency of ORF1p, leading to reducing LINE-1 ribonucleoprotein particles.^79^ Furthermore, YTHDC1 acts as a repressor of the 2C-like induction program,^80^ relies on its m^6^A-binding activity to facilitate KAP1 recruitment to LINE1 scaffold targets, and acts upstream of SET domain bifurcated histone lysine methyltransferase 1 (SETDB1) to co-regulate the silencing of LINE-1 via the decrease of H3K9me3, thereby maintaining ESC identity and restricting the transition back to the 2C-like state.^81^

Interestingly, the HUSH and nuclear exosome complexes control the expression of TE transcripts at the transcriptional or post-transcriptional level in mammalian cells, respectively, with LINE-1 manifestation being the predominant category.^82^^,^^83^ Nuclear exosomes have been shown to play an important role in the processing and decaying of various RNAs. Correspondingly, “nuclear exosome targeting (NEXT)” promotes m^6^A-modified LINE-1 RNA decay,^75^ while YTHDC1 also triggers its decay in mESCs via NEXT, primarily inhibiting their expression by affecting shorter non-polyadenylated transcripts.^82^ Notably, transcripts of the LINE-1 retrotransposon are also targets of *Zcchc8*, a central factor of the NEXT complex, as well as being driven for degradation in ESCs. Consistently, higher levels of LINE-1 RNA and more accessible chromatin conformations were observed in Zcchc8-deficient oocytes and embryos.^84^ This suggests that NEXT-associated LINE-1 is of great significance for post-transcriptional regulation of early embryonic development and pluripotency.

#### Histone modification and heterochromatin remodeling of LINE-1

Importantly, the stage-specific activation of retrotransposons depends on precise epigenetic regulation during early human development. Histones undergo numerous post-translational modifications (PTMs), which play a critical role in regulating chromatin structure and DNA-templated processes as epigenetic marks.^85^ The landscape of histone PTM is regulated by interconnected signaling pathways involving writers that catalyze the formation of specific PTMs, readers that recognize particular PTMs, and erasers that remove PTMs. In addition to transcription, these PTMs serve as fundamental epigenetic regulators that control crucial cellular processes, including DNA repair and DNA replication.^86^ During this process, H1 linker histones cooperate directly with suppressor of variegation 3–9 homolog 1 (Suv39h1), suppressor of variegation 3–9 homolog 2 (Suv39h2), and SETDB1 to mediate the repressive H3K9me3 in mESCs, thus profoundly de-repressing the transcription of LINE-1 and ERVs.^87^

Remarkably, ubiquitination may also play a role, as a recent study has shown that the E3 ligase tripartite motif containing 41 (TRIM41) interacts with and ubiquitinates ORF2p, thereby affecting its stability, whereas cyclic GMP–AMP synthase (cGAS) enhances the binding of ORF2p to TRIM41, thereby promoting TRIM41-mediated degradation of ORF2p and inhibiting LINE-1 reverse transcription.^88^

The HUSH complex performs a central role through epigenetic regulation and is responsible for silencing TEs.^82^ Both transcription activation suppressor (TASOR) and M-phase phosphoprotein 8 (MPP8), components of HUSH, are tightly associated with LINE-1, the former being involved in the SETDB1-dependent regulation of the H3K9me3 marker on the LINE-1 loci.^83^ Significantly, the C-terminal portion of MPP8 is essential for mESC self-renewal, the lack of which results in unstable MPP8 chromatin binding and increased expression of LINE-1.^89^ Furthermore, the HUSH complex can also act in conjunction with DNA polymerase ε to promote the asymmetric distribution of H3K9me3 thereby silencing LINE-1 expression during DNA replication.^90^ Intriguingly, while both NEXT and HUSH function in a pleiotropic manner, full-length LINE-1 is nevertheless up-regulated when HUSH is depleted because it “circumvents” NEXT-dependent decay.^82^ These have shown us the fascinating diversity of mechanisms we have uncovered for the involvement of the HUSH complex in the inhibition of LINE-1 elements.

Taken together, the transposition function of LINE-1 is dependent on the reverse transcriptional process of LINE-1 RNA, which is strictly regulated by m^6^A modifications. It is worth considering that m^6^A modifications on different types or structures of LINE-1 RNAs contribute to the variance, in addition to that these investigations have mainly focused on ORF1p, with the effect on the enzymatic activity of ORF2p, though, remains to be further elucidated. Evidently, understanding RNA modification is helpful to clarify the mechanisms of LINE-1 in preimplantation development.

#### Highly coordinated molecular mechanism to keep LTRs under tight control

Moreover, due to the relief of global DNA methylation repression, LTRs are activated and the subsequent repression coincides with the gradual reestablishment of H3K9me3 mediated by the modifier Suv39h1/h2 and ERG-associated protein with SET domain (ESET) complexes.^91^ A similar process involves the histone chaperone chromatin assembly factor 1 (CAF-1).^92^ Both murine leukemia virus (MLV) and IAP ERVs became demethylated in mouse embryos targeted for knockout of both alleles of the DNA methyltransferase 1(DNMT1), and IAP transcripts were expressed up to 100-fold relative to wild-type embryos. IAPs are most potently inhibited via the SETDB1-KAP1 (also named TRIM28, tripartite motif containing 28) silencing complex.^93^ The study further demonstrates that the scaffold protein KAP1 acts as a recruitment factor for the SETDB1-KAP1 complex and is also required for the silencing of ERVs.^94^ Histone demethylase lysine-specific demethylase 1A (KDM1A/LSD1) is required for silencing of a subset of class III ERVs by removal of H3K4me1/me2 targeting MERV-L sequences or LTR elements.^95^^,^^96^ Studies in mESCs have suggested that histone methyltransferase Suv39h1/h2 is also responsible for ERV silencing by establishing H3K9me3.^97^ In addition, H3K27me3 could also regulate the silencing of a distinct set of LTRs in early embryos. According to recent studies, chromatin assembly factor 1 subunit A (CHAF1A) functions as the core factor in H3K9me3-mediated LTR silencing in early embryos, and was essential for proper development to the blastocyst, and may be a prerequisite for the totipotency-to-pluripotency transition.^91^ RNA methylation also plays roles, and m^6^A has a protective effect in maintaining cellular integrity by clearing reactive ERV-derived RNA species.^76^ H3K9me2/me3 is associated with transcriptional repression and has a major role in ERV repression in mice.^27^^,^^94^^,^^98^

### TEs play a versatile role in preimplantation development

Retrotransposons play critical roles during preimplantation embryogenesis, including ZGA, totipotency, pluripotency, and lineage specification. Epigenetic remodeling during preimplantation development may contribute to genomic instability. Throughout this process, as mentioned previously, retrotransposons are either activated to function as a gene regulator or suppressed by the host cell to prevent widespread and detrimental reverse transcription. Subsequently, the host employs their abundant functional pool in various ways, including cis-regulation, epigenetic regulation, non-coding RNAs, and functional protein complexes. We provide an overview of the epigenetic- and transcription-based regulation of retrotransposons in early embryo development here and discuss the potential relationship between retrotransposon expression and genomic instability occurring at such stages.

#### LINE-1 elements remodel histone modifications

Advances in low-input or single-cell chromatin analysis have illuminated specific aspects of chromatin. The PTMs regulate numerous well-established cellular processes, and mounting evidence suggests that epigenetic regulatory mechanisms are essential for the precise spatiotemporal orchestration of gene expression, ensuring the tightly controlled molecular dynamics necessary for proper early embryonic development. Maternal and paternal H3K4me3 are regulated by lysine demethylase 5A/5B (KDM5A/5B). At the 2-cell stage, broad H3K4me3 is reprogrammed from gametes to embryos in a ZGA-dependent manner, with signaling restricted to the region of the transcription start site.^99^ In mouse preimplantation development, the sperm H3K27me3 is removed, with non-promoter region domains of H3K27me3 remaining transiently in embryos before blastocyst, after which canonical H3K27me3 is established at promoters in blastocyst.^100^ In particular, H3K4me3 and H3K27me3 established bivalence at the developmental gene promoter of embryonic day 6.5 epiblast.^100^ Similarly, the global H3K27me3 under the responsibility of polycomb repressive complex 2 (PRC2) of human embryos is thoroughly erased by the 8-cell stage (the beginning of ZGA) and reestablished in the morula stage.^101^ Both modifications above also have bivalent marks by co-occupancy, which widely exist in gametes, somatic cells, and primordial germ cells,^102^ rather than preimplantation embryos in mice and human,^103^ raising the possibility that they may predominantly function during exit from totipotent. In mice, the temporal transition of H3K36me3 catalyzed by SET domain containing 2 (SETD2) from the parental to the zygotic mode largely coincides with that of ZGA.^104^ These findings indicate that there are not only distinct roles but also intricate cross-talks between diverse histone modifications in mouse and human preimplantation development.

Retrotransposons are considered a rich source of noncoding regulatory elements that facilitate the production of TE chimeric transcripts during preimplantation development, and extensive epigenetic reprogramming of retrotransposons is integral to the developmental program. For instance, the activation of LINE-1 regulates global chromatin accessibility.^5^ In both hESCs and mESCs, the early embryonic control of LINE-1 is an evolutionarily dynamic process that occurs in different corresponding ancestral genomes at different historical periods.^68^ LINE-1 is expressed in 2-cell embryos and enhances chromatin accessibility. The subsequent depletion of LINE-1 after ZGA is imperative for the continuation of developmental progression.^105^ Conversely, the premature suppression of LINE-1 elements leads to decreased chromatin accessibility, while an extended activation of LINE-1 prevents the gradual chromatin compaction that occurs naturally in developmental progression.^5^ Fascinatingly, the developmental arrest caused by the extension of LINE-1 transcription is not associated with the reverse transcriptase activity possessed by ORF2p, suggesting that the primary function of LINE-1 is at the chromatin level.^5^

Specifically, H3K4 demethylation and H3K9 methylation are involved in the transcriptional switch at the 2-cell stage that histone-based defense mechanisms protect the genome from LINE-1 retrotransposition during pre-implantation embryo development.^106^ *Dppa2* and *Dppa4* serve as important safeguards of focal epigenetic states, which play non-redundant roles in preserving a targeted subset of developmentally relevant promoters and preserving LINE element activation in mESCs by acquiring *de novo* DNA hypermethylation. In their absence, developmental genes in pluripotent cells and young LINE-1 elements that bind specifically to *Dppa2* lose H3K4me3 and acquire ectopic DNA methylation.^107^ In addition, LINE-1 is expressed throughout development before embryo implantation, which may be as much about the epigenetic capacity of the *Dppa2/4*-dependent system to maintain developmental promoters as it is about escaping epigenetic silencing at the pluripotent stage.^107^ Otherwise, three key acetylated residues in the histone core, H56K3ac, H64K3ac, and H122K3ac, produce different dynamics at different developmental orders, with the most significant changes in H56K3ac. In the hESC model, both H3K56ac and LINE peak at the 2-cell stage, and there are abundant H3K56ac reads at LINE loci and there is a negative correlation between H3K56ac enrichment and evolutionary ages of LINE members.^108^ Therefore, LINE-1 is integral to the histone modification in preimplantation.

#### Modulation of early development by LINE-1 through cis- and trans-transcriptional activation

Extensive investigations of cis-regulatory sites and trans-acting factors have mechanistically revealed the important role exerted by LINE-1 on development. Numerous young TEs have deposited cis-regulatory elements that are active in early embryonic cells. A subset of young LINE-1 5′ UTRs, known as L1Md_Ts, have been demonstrated to function in cis to regulate gene expression as enhancers in the naïve state, in which L1Md_T functions as an enhancer. Elongation factor for RNA polymerase II 3 (ELL3) can regulate naïve-primed transition through the L1Md_T-AKT serine/threonine kinase 3 (AKT3)-extracellular signal-regulated kinase (ERK) pathway.^69^ The trans-acting functions of LINE-1 are also crucial for modulating cellular identity during early development.

#### LTR retrotransposons in early embryonic development

MERV-L elements are used as alternative promoters to facilitate the transcription of a substantial number of genes specific to preimplantation.^44^ LTR elements harbor a Dux binding motif, which initiates the activation of the MERV-L family and defines the cleavage-specific transcriptional programs in mice and humans.^43^, ^44^, ^45^ Similarly, DUX4 as one of the drivers exactly induces the expression of ZGA genes in hESCs, suggesting that retrotransposons have been co-opted as gene regulatory regions during evolution. LTRs are able to transcribe neighboring ZGA-associated genes, resulting in “chimeric” LTR–host transcripts.^59^^,^^109^ For example, several host genes such as *Tcstv1*, *Tcstv3*, *Ubtfl1*, *Chit1*, *Eif1a*, and *Zfp352* have transcripts beginning from LTR elements in mESCs.^96^ In addition, pluripotent transcription factor (Oct4, Nanog, Klf4, and Lbp9) binding sites in HERV-H within the LTR can also induce ERV expression, which is a hallmark of naive-like hESCs.^110^ ERV elements also can be involved in organizing high-order chromatin structure after fertilization, thereby facilitating the global remodeling of chromatin architecture during preimplantation development.^111^ However, even in the absence or mutation of the corresponding TF, the host promoter retains the ability to activate ZGA, which means that alternative additional transcription factor regulation can compensate for this role, as exemplified by the Dppa family.^112^

In particular, ERVs are able to escape their DNA methylation and transcription through TET activity in preimplantation embryos,^95^ and DNA demethylation of retrotransposons during this process results in their activation at the 2-cell stage, where numerous ERVs (MERV-L and ETn/MusD) are derepressed and expressed at extremely high levels.^59^^,^^113^^,^^114^

As the overall DNA methylation level decreases during preimplantation embryonic development, the epigenetic silencing factors on LTRs switch from DNA methylation to histone modifications in response to several factors, with H3K9me3 playing a major role. Specifically, the rebuilding of H3K9me3 on LTRs is involved in silencing their active transcription triggered by DNA demethylation in preimplantation development.^115^ The drastic reprogramming of H3K9me3-dependent heterochromatin upon fertilization removes H3K9me3 barriers from promoter regions, creating a less constrained epigenetic environment for subsequent ZGA and may be required for the embryo to reach the totipotent state. H3K9me2/me3 catalyzed by histone methyltransferase SETDB1 is enriched at the LTRs of many ERVs in mESCs, and knockout of SETDB1 from mESCs can significantly up-regulate several ERVs.^27^

It has been shown that H3K27me3 is associated with silencing of MLV and IAP ERVs in mouse ES cells,^116^ and mutations in KDM1A cause abnormal expression of MERV-L in mice, and lead to increased methylation of H3K4, increased acetylation of H3K27 and decreased methylation of H3K9, and even embryonic developmental arrest.^96^ In contrast, unregulated ERV-mobilised transposition activities can cause insertional mutations or chromosomal abnormalities, therefore, ERVs should be silenced through DNA methylation in terminally differentiated somatic cells where otherwise aberrant activation threatens genomic integrity, leading to cancer and autoimmune disorders.^117^^,^^118^ As exemplified by elevating HERV transcripts and protein expression that have been detected in human patients have been shown to be associated with the etiology of a number of diseases, such as breast cancer, autoimmune diseases, and neurological disorders.^14^^,^^119^ Besides the effects mentioned above, integrating a HERV also can inactivate or eliminate control of an essential gene or enable homologous recombination, resulting in a variety of diseases.^120^

### LINE-1 regulates the exit from totipotency

Totipotency refers to the ability to generate a complete organism, including both embryonic and extra-embryonic tissues. This ability is exhibited exclusively by the zygote and early-stage embryos (2-cell stage in mice and 8-cell stage in humans).^121^ The regulation of totipotency remains unclear due to ethical issues, the limited cells derived from early-stage embryos, and the lack of *in vitro* models. A subpopulation of ESCs that closely resemble the 2-cell stage embryo molecularly and epigenetically, termed 2-cell-like ESCs, provides a valuable *in vitro* model for studying early development and totipotency.^58^ Additionally, numerous studies have generated totipotent stem cells with characteristics of 2-cell embryos, such as totipotent blastomere-like cells,^54^ totipotent-like stem cells,^122^ and chemically induced totipotent stem cells.^123^ Correspondingly, the importance of *in vitro* cell models that reproduce the early human blastocyst stage becomes self-evident. Several studies have reported the development of human 8-cell-like cells from pluripotent stem cells and have characterized their cellular properties at the single-cell level.^124^, ^125^, ^126^, ^127^

The study of mammalian totipotency has emerged as a pivotal field within developmental biology, particularly with the transposon-mediated regulation of gene expression. According to the systematic analysis of the global transcriptome of repetitive elements in early embryos, many retrotransposons are expressed during the initial transcriptional activation of the zygotic genome, mainly including ERVs, LINE-1, SINEs, and IAP.^5^^,^^47^ The typical example of TEs is developmentally regulated and utilized as alternative promoters and first exons for host genes, regulating their expression in oocytes and cleavage. In addition, TEs can trigger the reprogramming of embryonic genes affecting the transition from oocyte to embryo and pre-implantation embryos.

Mounting evidence has identified LINE-1 as a key totipotency-related repeat sequence that remains active in the human genome and displays an unusual pattern of evolution in early development.^128^ Studies *in vivo* using mouse models have shown that LINE-1 activation and expression is one of the earliest transcriptional events emerging during ZGA at the 2-cell stage.^105^ Of note, the trans-acting functions of LINE-1 are crucial for the modulation of exit cell totipotency.^129^ Inactivation of LINE-1 expression leads to developmental arrest in 2-cell embryos.^130^ In ES cells, this results in defective self-renewal, which may be the result of a combination of reduced *de novo* transcription (*e.g.*, rRNA) and cell-cycle deficits.^21^ While LINE-1 deficiency does not induce precocious differentiation, LINE-1 represses MERV-L and 2C in ESCs through a dependence on the Dux transcriptional programmes. The combination of reduced nascent transcription (*e.g.*, rRNAs) and cell cycle defects LINE-1 RNA has a sustained requirement for inhibition of 2C programming and developmental progression. Mechanistically, LINE-1 expression regulates exit from the 2-cell state by repressing the 2C program induced by Dux, along with nucleolin and KAP1/TRIM28.^21^ Moving to a more posterior developmental stage, the enhancer function of L1Md_T controlled by ELL3 also promotes naïve-primed transitions.^69^ The observed developmental phenotypes do not imply the coding nature of the transcript and retrotransposition. Furthermore, LINE-1 ORF1p is predominantly cytoplasmic, in contrast to the nuclear localization of LINE-1 RNA.^21^

However, further investigation is required to determine whether this is also the case in early embryos. LINE-1 is essential to orchestrate developmental progression during pre-implantation and for the self-renewal of ESCs. Nevertheless, from the previously mentioned current advances, it is less associated with retrotransposon function and more chromatin-associated nuclear effect, since inhibition of RT activity of LINE-1 does not rescue embryos from arresting development.^5^ Collectively, LINE-1 is integral to the transcriptional circuitry in early development. However, their exact roles in ZGA, totipotency, pluripotency, and lineage commitment await further investigation, and much remains to be done to understand how TEs are regulated in the totipotency of early development.

### Highlights and future perspectives

From an evolutionary perspective, the beginning of LINE-1 is generally considered detrimental. They are transcriptionally silenced through the acquisition of heterochromatin signatures in the majority of somatic cells.^60^ In contrast, LINE-1 is typically expressed throughout preimplantation development, which has generated significant interest in recent years. Although the retrotransposition of LINE-1 does not directly regulate 2C gene expression or ESC self-renewal, it plays a crucial role in shaping the chromatin landscape of the early development process.^21^ The evolution of the mechanisms by which LINE-1 controls developmental potency is a subject of interest. Remarkably, due to their rapid evolution and the repeated nature, TEs do not tend to be fragile and vulnerable, instead, they may be robust and adaptive. Understanding the specificity function of TEs in early embryo development offers a novel perspective for investigating the molecular mechanism in totipotency.

Despite their potential functionalities in embryogenesis, LINE-1 can be dysregulated in several pathological conditions, such as aging and cancer. Aging is associated with extensive remodeling of the epigenome. Numerous studies have provided evidence that reduction in heterochromatin contributes to an increase in retrotransposon activity, potentially leading to cellular senescence^131^. For instance, during aging and DNA damage, *SIRT6* is depleted from LINE-1 loci, facilitating the activation of LINE-1 that was previously silenced.^132^ Additionally, the activation of the interferon type I (IFN-I) response, which occurs concurrently with age-associated inflammation, is triggered by the reverse transcriptase activity of LINE-1.^133^ Subsequently, LINE-1 is also critical for telomere maintenance in telomerase-positive tumor cells and may be a target in the rational treatment.^134^ In addition, LINE-1 also affects the function of immune cells, potentially regulating T-cell dysfunction.^135^ It is well known that LINE-1, the only autonomously moving transposon in humans, may challenge genomic stability by inducing insertion mutagenesis. With some exceptions, however, a recent study has shown a potential role for LINE-1 in inhibiting tumor progression, with the methyl H3K9-binding protein MPP8 inhibiting LINE-1 in myeloid leukemia, and reactivation of LINE-1 impairing leukemia cell growth *in vitro* and xenografts.^136^

Recent advances have updated the view of retrotransposons. However, the potential mechanisms of TEs in early human embryonic development, human aging, inflammation, and cancer are still obscure. Further research is necessary to gain a better understanding of the specific functions and mechanisms of LINE-1, particularly in early embryonic and totipotency transition.

## Conclusion

The intricate involvement of TEs, particularly LINE-1, in early embryonic development underscores their pivotal role. Their dynamic expression and multifaceted contributions illuminate the regulatory complexity vital for developmental progression and totipotency, offering valuable insights into early human embryonic development and associated diseases.

## CRediT authorship contribution statement

**Ru Ma:** Writing – original draft, Visualization. **Nan Xiao:** Writing – review & editing. **Na Liu:** Conceptualization, Funding acquisition, Supervision, Writing – review & editing.

## Conflict of interests

The authors have no competing interests to declare.
